# Ablation of idiopathic ventricular arrhythmias

**DOI:** 10.1007/s12471-018-1095-3

**Published:** 2018-02-26

**Authors:** J. R. de Groot

**Affiliations:** Heart Center, Department of Cardiology, Academic Medical Center, Amsterdam, The Netherlands

Idiopathic ventricular arrhythmias (IVA) is a term commonly used to describe premature ventricular complexes (PVCs) or ventricular tachycardias (VT) in the absence of structural heart disease. Most IVA (approximately 70%) arise from the right ventricular outflow tract (RVOT) and can be recognised on the ECG by a left bundle branch morphology of the QRS complex, an inferior axis, and an R/S transition, usually at V4. Other sites of origin of IVA include the aortic cusps, the left ventricular outflow tract (often characterised by an earlier R/S transition in the precordial ECG leads and a left or right bundle branch block morphology), the great cardiac veins, the epicardial myocardium, the aorta-mitral continuity or rarely from the pulmonary artery. For daily clinical practice, idiopathic ventricular arrhythmias need to be discriminated from those associated with structural heart disease, arrhythmogenic right ventricular cardiomyopathy (ARVC) in particular. Aside from the procedural consequences the latter diagnosis has, there are obvious differences in prognosis, and prevention of sudden cardiac death may be warranted. ECG characteristics can indicate the presence of ARVC. Aside from T‑wave inversion in V1–3, the QRS is wider during sinus rhythm, the upstroke of the S‑wave duration is longer, the duration of the QRS is longer in V1–3 than in V4–6 as is the JT-interval in ARVC patients than in patients with idiopathic RVOT VT[1]. The clinical characteristics of middle-aged or elderly patients with ARVC, who are more frequently recognised in the era of molecular genetic testing, have been described recently with depolarisation changes and structural alterations as most outstanding findings [2]. Contrary to ARVC-related ventricular arrhythmias, idiopathic ventricular arrhythmias generally have a favourable prognosis, but life-threatening events have been described [1, 3]. Therefore, in the absence of structural heart disease, there are two indications for the treatment of ventricular arrhythmias. First and foremost, symptoms associated with arrhythmias (mostly palpitations) form an indication for treatment with medication or catheter ablation. Second, diminished left ventricular function resulting from a high burden of ventricular arrhythmias or from incessant high rates (tachycardiomyopathy) may indicate treatment. Of note, the patient’s symptoms may relate to both the arrhythmia itself and to the resulting diminished left ventricular function. Therefore, the guidelines give a class 1, level of evidence B recommendation for ablation of RVOT arrhythmias in symptomatic patients, in patients in whom a trial with antiarrhythmic drugs was unsuccessful, or patients with a decline in left ventricular function due to the burden of ventricular arrhythmia [4]. For ventricular arrhythmias arising from the LVOT, epicardium or aortic cusps, there is a class 1, level of evidence C recommendation to treat with class 1C antiarrhythmic drugs. Catheter ablation should only be performed in these patients after antiarrhythmic medical treatment fails (class 2A, level of evidence B) [4].

In this issue of the Netherlands Heart Journal, Oomen et al. present an observational, single high-volume centre study on 5 years of experience with catheter ablation of idiopathic PVCs or VTs [5]. They describe 131 patients (64% female, 99 (76%) with PVCs, 32 (24%) with idiopathic VT) who underwent a total of 147 ablation procedures between 1 January 2011 and 31 December 2015. Eighteen patients had a left ventricular ejection fraction lower than 50% prior to the ablation procedure. In all patients an ischaemic origin was excluded by coronary angiography or treadmill testing.

A total of 118 patients underwent a single procedure, in 11 patients two procedures were performed and in 2 patients three and four procedures, respectively. Eight patients (6%) had undergone a previous ablation procedure before the study period. The majority of IVA originated from the RVOT (60%); 19% arose from the aortic cusps. Procedural success, which the authors define as elimination and non-inducibility of the clinical arrhythmia, was achieved in 89% of procedures, whereas in six procedures (4%) it could not be determined due to lack of PVCs at the start of the procedure. Sustained successful ablation, defined as a reduction of PVCs by ≥80% and elimination of VTs after 3 months, was documented in 82% of the 90 patients in whom repetitive Holter monitoring was available. The median burden of PVCs decreased from 15.5% (25.5% in patients with decreased left ventricular function) to 0.1% in patients with either procedural or sustained success. In patients with diminished left ventricular function, the ejection fraction normalised after sustained successful ablation (15 patients); in 3 patients without sustained ablation success, the left ventricular function did not change. Interestingly, there was no difference in mean heart rate before and after the ablation in these 18 patients, indicating that tachycardiomyopathy is an unlikely explanation for the left ventricular failure. Major complications, consisting of tamponade (*n* = 3), abdominal bleeding (*n* = 1) and third-degree AV block (*n* = 1) occurred in 3.8% of patients.

Hence, ablation of idiopathic ventricular arrhythmias results in a massive reduction of PVC burden and VT in the vast majority of patients with a low, but not negligible risk of procedural complications.

As the endpoint of the study was defined as sustained ablation success at 3 months after the procedure, it is not surprising that the mean follow-up in this study was less than one year. However, as the authors mention as well, since the procedure is aimed at reducing symptoms, it may be expected that patients with recurrent symptoms would return to their clinic at some time point. In the absence of longer follow-up, however, the statement that ablation of IVA is curative may be somewhat overstated. This is further stressed by the notion that both procedural success and sustained success are determined from a relatively narrow monitoring frame, that is, the duration of the procedure and a 24-hour Holter. As the occurrence of IVA can be variable over time (and indeed no PVCs were encountered in 6 patients during the procedure), it is not possible to truly assess changes in PVC burden without continuous monitoring. Nevertheless, the reduction of PVC burden on 24-hour Holter by a factor of >150 is convincing enough. Hence, Oomen et al. show that in experienced hands ablation of IVA is an effective procedure. Invasive treatment of IVA, as predominantly indicated for symptoms, seems an ideal case for shared decision-making with the patient, in which the balance between chance of success and risk of harm can be appreciated in a patient-tailored manner.
